# A machine learning-based predictor for the identification of the recurrence of patients with gastric cancer after operation

**DOI:** 10.1038/s41598-021-81188-6

**Published:** 2021-01-15

**Authors:** Chengmao Zhou, Junhong Hu, Ying Wang, Mu-Huo Ji, Jianhua Tong, Jian-Jun Yang, Hongping Xia

**Affiliations:** 1 Department of Anesthesiology, Pain and Perioperative Medicine, The First Affiliated Hospital of Zhengzhou University, Zhengzhou, 450000 China; 2 School of Medicine, Southeast University, Nanjing, 210009 China; 3 Department of Colorectal and Anal Surgery, The First Affiliated Hospital of Zhengzhou University, Zhengzhou, 450000 China; 4 Department of Pathology, School of Basic Medical Sciences, Sir Run Run Hospital, State Key Laboratory of Reproductive Medicine, Key Laboratory of Antibody Technique of National Health Commission, Nanjing Medical University, Nanjing, 211166 China

**Keywords:** Health care, Medical research

## Abstract

To explore the predictive performance of machine learning on the recurrence of patients with gastric cancer after the operation. The available data is divided into two parts. In particular, the first part is used as a training set (such as 80% of the original data), and the second part is used as a test set (the remaining 20% of the data). And we use fivefold cross-validation. The weight of recurrence factors shows the top four factors are BMI, Operation time, WGT and age in order. In training group:among the 5 machine learning models, the accuracy of gbm was 0.891, followed by gbm algorithm was 0.876; The AUC values of the five machine learning algorithms are from high to low as forest (0.962), gbm (0.922), GradientBoosting (0.898), DecisionTree (0.790) and Logistic (0.748). And the precision of the forest is the highest 0.957, followed by the GradientBoosting algorithm (0.878). At the same time, in the test group is as follows: the highest accuracy of Logistic was 0.801, followed by forest algorithm and gbm; the AUC values of the five algorithms are forest (0.795), GradientBoosting (0.774), DecisionTree (0.773), Logistic (0.771) and gbm (0.771), from high to low. Among the five machine learning algorithms, the highest precision rate of Logistic is 1.000, followed by the gbm (0.487). Machine learning can predict the recurrence of gastric cancer patients after an operation. Besides, the first four factors affecting postoperative recurrence of gastric cancer were BMI, Operation time, WGT and age.

## Introduction

The global incidence of gastric cancer is the fourth in malignant tumors and the second in mortality. There are nearly 1 million cases of new gastric cancer worldwide each year, of which nearly 50.0% occur in China. The prognosis of early gastric cancer is good, but the clinical symptoms are atypical and the signs are not obvious. The postoperative recurrence rate (40.0–70.0%) remained high^1^. It has been reported that the average time to recurrence of gastric cancer was 20.5–28.0 months after operation^2^. However, when gastric cancer recurs after the operation, some chemotherapy and immunotherapy can be used in close cooperation to control cancer and reduce necrosis, to create conditions and strive for operation.

In recent years, big data and machine learning have led to innovative changes in many industries. With the development of precision medicine plan, the combination of health and medical big data and machine learning brings people the imagination space of the future big data health cause. The method of machine learning is especially suitable for prediction based on existing data. By capturing complex nonlinear relations in the data, the machine learning algorithm can improve the accuracy of prediction more than the conventional regression model. At present, machine learning can predict the survival of breast cancer patients at an early stage^3^. Using machine learning technique can predict the malignant degree of breast lesions^4^; Machine learning was used to better predict early biochemical recurrence^5^; Machine learning is a branch of artificial intelligence, which has been used in tumor risk assessment, lesion detection, prognosis prediction and treatment response^6^; Deep learning can effectively solve medical problems that were previously thought unsolvable^7^.

In conclusion, postoperative recurrence of gastric cancer are key factors affecting the prognosis of gastric cancer, and it is very important to actively explore the adverse factors affecting postoperative recurrence of gastric cancer to detect and evaluate the recurrence or metastasis of postoperative gastric cancer. Therefore, we used a machine learning technique to predict the tumor recurrence of gastric cancer patients after operation.

## Materials and method

### Data source

Data is available at BioStudies database, accession numbers: S-EPMC4344235. This study included 2012 patients.

Data from the retrospective studies included age, gender, pathological characteristics, treatment-related factors, and the follow-up period related to survival status.

### Machine learning algorithm

Logistic regression, a kind of generalized linear regression analysis model, is often used in such fields as automatic disease diagnosis and economic prediction.

The decision tree algorithm belongs to the category of supervisory learning.

In machine learning, a random forest is a kind of classification/regression which contains multiple decision trees (CART tree). The final classification result is decided by each decision tree vote/ average, that is, a few obey the principle of most. The stochastic forest in turn corresponds to the fusion of the model of several decision trees (CART trees).

The GBDT is also called MART. It is an iterative decision tree algorithm. The trees in GDBT are all regression trees. Only the accumulation of the results of regression trees is meaningful, and the addition of the results of classification is not meaningful.

The light GBM (light gradient boosting machine) is a framework to implement GBDT algorithm, which supports efficient parallel training.

### Data processing

Data were processed in R (3.5.3) language. P < 0.05 was taken as the difference with statistical significance; Multiple imputations were used for missing variables. The machine learning was analyzed by python (3.6.5). The total population was randomly divided into a training group and test group according to the ratio of 8:2. The available data is divided into two parts (sometimes called training-test segmentation). In particular, the first part is used as a training set (such as 80% of the original data), and the second part is used as a test set (the remaining 20% of the data). Then, a prediction model is established by using the training set. To get the best model, manual parameter adjustment and grid search are used. And we use fivefold cross validation^8–10^. Then the trained model is applied to the test set for prediction. Choose the best model according to its performance on the test set^11^. And the data are normalized by us. The parameters of the machine learning model are shown in Supplementary Table 1.

**Table 1:** Baseline data.

RECURR	No	Yes	P-value
N	1607	405	
Age (year)	58.5 ± 11.5	58.5 ± 12.2	0.697
Weight (kg)	61.4 ± 10.2	60.0 ± 10.4	0.011
Height (cm)	162.2 ± 8.4	162.3 ± 8.4	0.982
BMI (kg/m^2^)	23.3 ± 3.2	22.8 ± 3.1	0.004
Operation time (min)	168.6 ± 52.0	183.3 ± 55.2	< 0.001
Tumor size (cm)	4.3 ± 2.8	6.7 ± 3.4	< 0.001
**Sex**			0.980
Male	1110 (69.1%)	280 (69.1%)	
Female	497 (30.9%)	125 (30.9%)	
**Location**			< 0.001
Upper	234 (14.6%)	65 (16.0%)	
Middle	499 (31.1%)	108 (26.7%)	
Lower	860 (53.5%)	204 (50.4%)	
Whole	14 (0.9%)	28 (6.9%)	
**Extent of LN dissection**			0.409
D2	131 (8.2%)	28 (6.9%)	
D1 plus	1476 (91.8%)	377 (93.1%)	
**Chemotherapy**			< 0.001
NO	950 (59.1%)	89 (22.0%)	
YES	657 (40.9%)	316 (78.0%)	

### Ethics approval

Because this is only a secondary data analysis study using public databases, there is no need to apply for ethics^25^.

## Results

### Correlation analysis and feature analysis

Comparison of basic indicators of patients in the two groups: there was no statistically significant difference in age and height between the two groups (P = 0.697 and P = 0.982). See Table 1.

**Figure 1:**
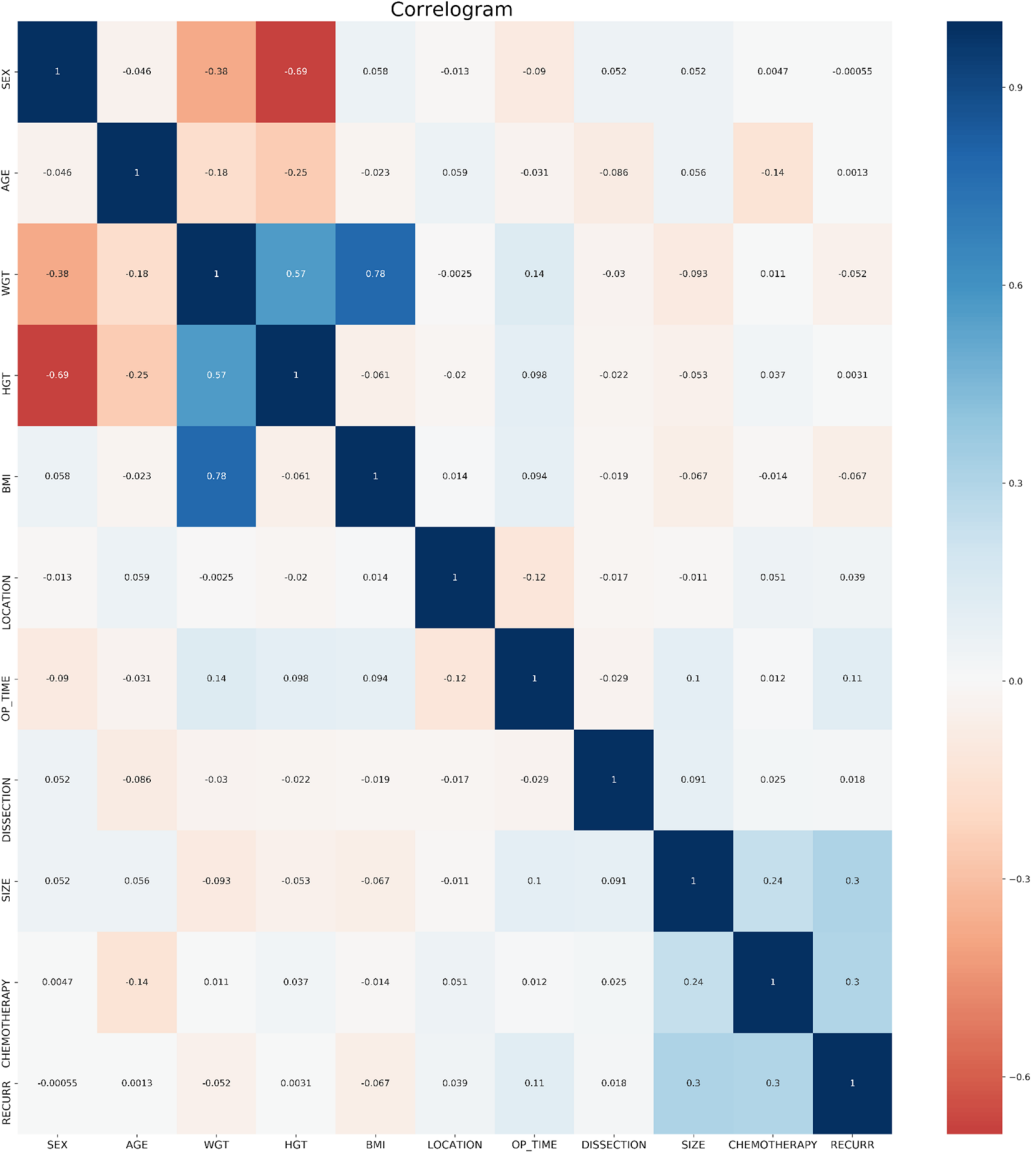
Correlation between variables.

**Figure 2:**
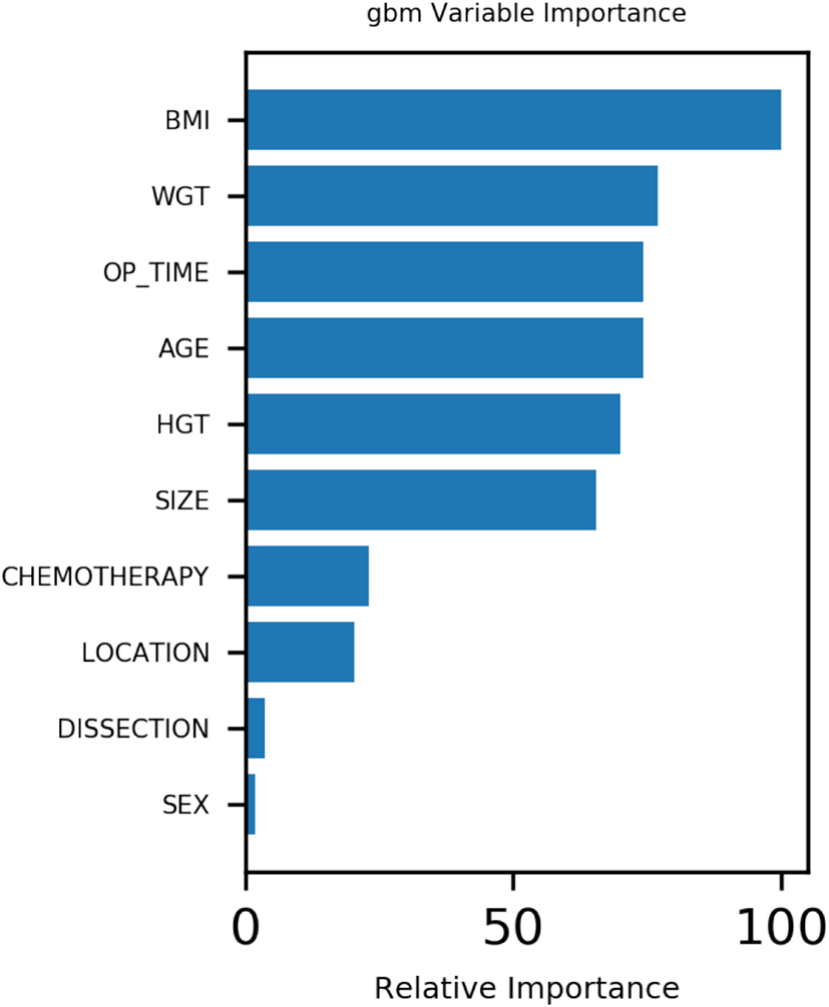
Variable importance of features included in the machine learning algorithm for prediction of recurrence of patients with gastric cancer after operation. *Note*: gbm: LightGBM.

The results of gbm algorithm showed that the first 4 factors were ranked in order: BMI, Operation time, WGT and age, respectively (Figs. 1 and 2).

### Training set results

**Figure 3:**
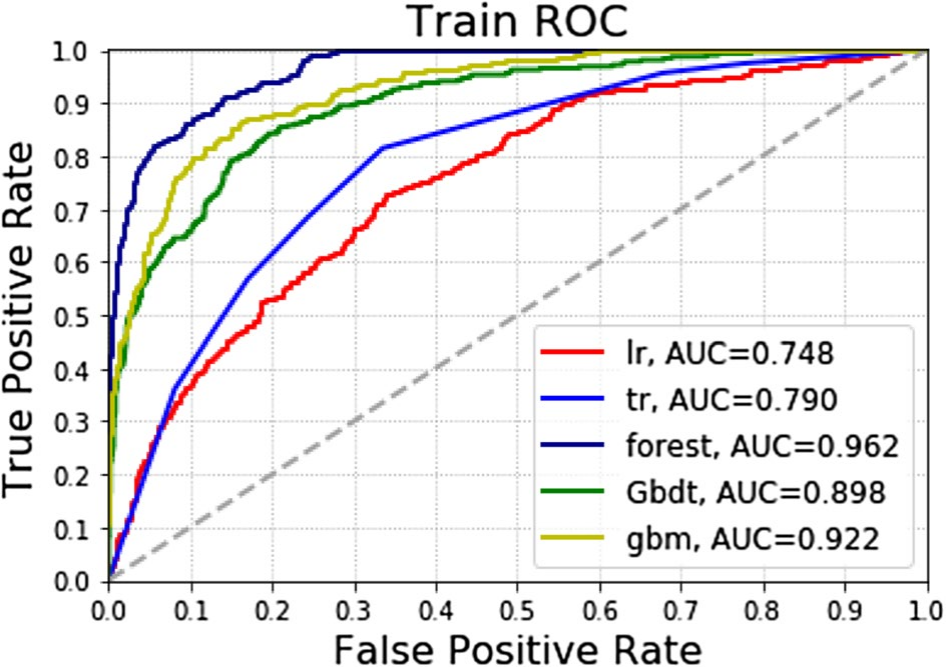
Different machine learning algorithms predict the recurrence of patients with gastric cancer after the operation in the training group.

In training group: among the 5 models, the accuracy of gbm was 0.891, followed by gbm algorithm was 0.876; The AUC values of the five algorithms are from high to low as forest (0.962), gbm (0.922), GradientBoosting (0.898), DecisionTree (0.790) and Logistic (0.748). The precision of forest is the highest 0.957, followed by the GradientBoosting algorithm (0.878).The recall rate for forest is up to 0.478, followed by the gbm (0.451) (Table 2 and Fig. 3).

**Table 2:** Forecast results for training group.

	Accuracy	Precision	Recall	AUC
Logistic	0.799	0.500	0.003	0.748
DecisionTree	0.807	0.529	0.361	0.790
Forest	0.891	0.957	0.478	0.962
GradientBoosting	0.868	0.878	0.401	0.898
gbm	0.876	0.869	0.451	0.922

### Test set results

**Figure 4:**
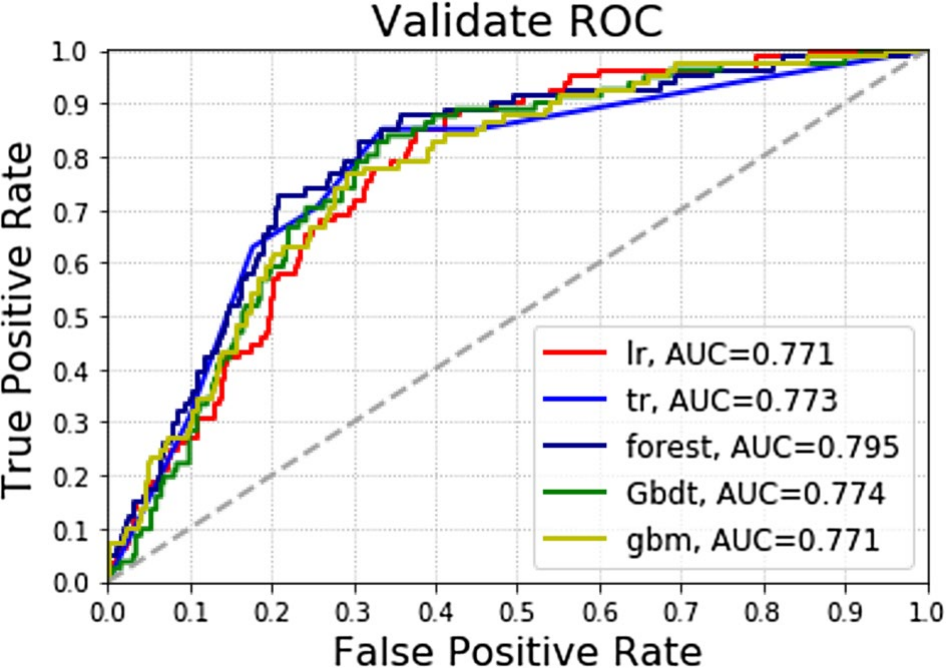
Different machine learning algorithms predict the recurrence of patients with gastric cancer after the operation in the test group.

In the test group: among the five algorithm models, the highest accuracy of Logistic was 0.801, followed by forest algorithm and gbm; The AUC values of the five algorithms are forest (0.795), GradientBoosting (0.774), DecisionTree (0.773), Logistic (0.771) and gbm (0.771), from high to low. The highest precision rate of Logistic is 1.000, followed by the gbm (0.487).The highest recall rate was 0.309 for the DecisionTree, followed by the gbm algorithm (0.235) (Table 3 and Fig. 4).

**Table 3:** Forecast results for testing group.

	Accuracy	Precision	Recall	AUC
Logistic	0.801	1.000	0.012	0.771
DecisionTree	0.782	0.439	0.309	0.773
forest	0.797	0.480	0.148	0.795
GradientBoosting	0.779	0.367	0.136	0.774
gbm	0.797	0.487	0.235	0.771

## Discussion

Currently, the treatment of early gastric cancer mainly involves an open operation. According to the location of gastric cancer and the size of lesions, the proximal or distal subtotal resection or total gastrectomy is selected. Mortality is very high in patients with gastric cancer due to its high morbidity and recurrence rate. Therefore, it is very important to find out the factors affecting recurrence and metastasis to reduce the mortality of gastric cancer. After identifying the influencing factors, correct and effective prevention methods are adopted to treat the patients, so that the postoperative recurrence rate can be effectively reduced and the postoperative quality of life can be greatly improved. The first 4 important factors affecting postoperative recurrence of gastric cancer were BMI, Operation time,WGT and age, respectively. And machine learning can predict the recurrence of gastric cancer after the operation.

The risk factors affecting the recurrence of gastric cancer are clinical, pathological and biomolecule. The later the clinical stage, the greater the probability of early recurrence, the shorter the survival time. Studies^12^ have shown that elevated early gastric cancer, infiltration depth to the submucosa, and concomitant lymph node metastasis are independent factors affecting postoperative recurrence of early gastric cancer. It was also found that age and macroscopic appearance of the tumor was associated with postoperative recurrence of early gastric cancer, while gender, tumor location, tumor size and histological type were not associated with postoperative recurrence of early gastric cancer^13^. Moriguchi et al.^14^ found that regional lymph node metastasis was a risk factor for gastric cancer recurrence. The results of this study were also similar.

Kattan et al.^14^ demonstrated a poor prognosis in the upper third of tumors. Our study also showed a correlation between tumor location and tumor recurrence.

Recently, studies have demonstrated the survival benefit of adjuvant chemotherapy after radical resection of gastric cancer^1^. However, in this study, chemotherapy was not negatively associated with tumor recurrence. This negative result may be influenced by the reason and the regimen and indication of adjuvant chemotherapy at each institution.

Bickenbach et al.^15^ concluded that high BMI was a postoperative complication of gastric cancer but not of long-term survival. Dhar et al.^16^ reported that high BMI was not conducive to the removal of gastric lymph nodes in 787 patients with gastric cancer. The results of Tokunaga et al.^17^ showed that the survival rate of patients with gastric cancer with high BMI was higher than that of patients with low BMI. Kruhlikava et al.^18^ concluded that BMI did not affect survival in patients with esophagogastric cancer. Migita et al.^19^ showed that underweight is a simple and reliable predictor of poor long-term prognosis in patients with gastric cancer. Kulig et al.^20^ reported that the median disease-related survival time of patients with high BMI was significantly longer than that of patients with low BMI. The results of this study showed that BMI was negatively correlated with tumor recurrence.

This study is addressed by the classification task. It should be to use accuracy (Ac), sensitivity (Sn), specificity (Sp) Matthews coefficient correlation (MCC) and AUC^21–23^. However, it appears that only 20% of the data correspond to the positive class. The imbalance (1:4) makes ROC, AUC-ROC, and especially accuracy, less useful in assessing the utility of a predictor. So we have adopted the AUC chart with the highest precision. And this imbalance in classification is normal in the application of machine learning-related medicine because the incidence and non-incidence of diseases are unbalanced.

Although machine learning has yielded good results in predicting the postoperative recurrence of gastric cancer, the present study has some limitations. Some patients were excluded due to lack of data, which may lead to selection bias. Besides, due to retrospective data, our study failed to refine the prediction of recurrence in some subgroups of the postoperative gastric cancer population, such as patients with gastric cancer combined with other malignant tumors and patients with gastric cancer with other special medical histories, which may cause some applicability of the study results. Further prospective studies on this aspect are needed in the future.

## Conclusion

To sum up, machine learning can predict the recurrence of patients with gastric cancer after an operation. Besides, the first four factors affecting postoperative recurrence of gastric cancer were BMI, Operation time, WGT and age.

## Supplementary information

Supplementary Table S1.

## Data Availability

Data is available at BioStudies database, accession numbers: S-EPMC4344235.
