# Supplementary material for: A machine learning-based predictor for the identification of the recurrence of patients with gastric cancer after operation
Source: Sci Rep. 2021 Jan 15;11:1571. doi: 10.1038/s41598-021-81188-6 (PMC7810757; doi:10.1038/s41598-021-81188-6)
Supplement: Supplementary file 1 — Supplementary Table S1. [file 41598_2021_81188_MOESM1_ESM.doc]

Appendix Table 1. Functions, Packages, and Tuning Parameters in the Anaconda Software Used for Each Machine Learning Algorithm

| Algorithm | Classifier | Package | Tuning Parameters |
| --- | --- | --- | --- |
| Logistic regression | LogisticRegression | from sklearn.linear_model import LogisticRegression | penalty='l2',tol=0.00001,C=0.1,fit_intercept=True,intercept_scaling=1,class_weight=None,max_iter=100,multi_class='ovr',verbose=0,warm_start=False,n_jobs=1 |
| DecisionTree | DecisionTreeClassifier | from sklearn.tree import DecisionTreeClassifier | splitter='best', max_depth=3, min_samples_split=80,min_samples_leaf =65, min_weight_fraction_leaf=0.01, max_features=None, random_state=42, max_leaf_nodes=None,class_weight=None |
| forest | RandomForestClassifier | from sklearn.ensemble import RandomForestClassifier | n_estimators=100,max_features = "auto",min_samples_leaf = 5 ,n_jobs = 1,random_state =42 |
| GradientBoosting | GradientBoostinglassifier | from sklearn.ensemble import GradientBoostinglassifier | learning_rate=0.1,n_estimators=100,max_depth=3, max_features='auto', min_samples_split=20,min_samples_leaf=3,random_state =42 |
| gbm | lgb.LGBMClassifier | lightgbm 2.2.0 | learning_rate=0.2, n_estimators=100, lambda_l1=0.2 ,lambda_l2= 2 ,max_depth=3, bagging_fraction = 0.6,feature_fraction = 0.6 |

Note:gbm: LightGBM
